# Dual solutions of nanomaterial flow comprising titanium alloy (Ti_6_Al_4_V) suspended in Williamson fluid through a thin moving needle with nonlinear thermal radiation: stability scrutinization

**DOI:** 10.1038/s41598-020-77996-x

**Published:** 2020-12-01

**Authors:** Umair Khan, A. Zaib, Ilyas Khan, Kottakkaran Sooppy Nisar

**Affiliations:** 1 Department of Mathematics and Social Sciences, Sukkur IBA University, Sukkur, 65200 Sindh Pakistan; 2 Department of Mathematical Sciences, Federal Urdu University of Arts, Science and Technology, Gulshan-E-Iqbal, Karachi, 75300 Pakistan; 3 Faculty of Mathematics and Statistics, Ton Duc Thang University, Ho Chi Minh City, Vietnam; 4 Department of Mathematics, College of Arts and Sciences, Prince Sattam bin Abdulaziz University, Wadi Aldawaser, 11991 Saudi Arabia

**Keywords:** Engineering, Mathematics and computing

## Abstract

Titanium alloy nanoparticle has a variety of applications in the manufacturing of soap and plastic, microsensors, aerospace design material, nano-wires, optical filters, implantation of surgical, and many biological treatments. Therefore, this research article discussed the influence of nonlinear radiation on magneto Williamson fluid involving titanium alloy particles through a thin needle. The arising system of partial differential equations is exercised by the similarity transformations to get the dimensional form of ordinary differential equations. The dual nature of solutions is obtained by implementing bvp4c. The study of stability has been carried out to check which of the results are physically applicable and stable. Influences of pertinent constraints on the flow field are discussed with the help of graphical representations and the method validation is shown in Table 1. The results imply that more than one result is established when the moving needle and the free-stream travel in the reverse directions. Moreover, the magnetic parameter accelerates the severance of boundary-layer flow, while the separation delays in the absence of the nanoparticle. The velocity gradient of nanofluid decays owing to the Williamson parameter in both branches of the outcome, while the temperature shrinks in the first or upper branch solution (stable one) and uplifts in the second or lower branch solution (unstable one). The size of the needle decreases the velocity in the upper solution and accelerates in the lower solution. The patterns of streamlines are more complicated due to the reverse direction of the free stream and thin needle.

## Introduction

A thin moving needle structure is explained as a paraboloid revolution regarding its direction of axes in addition to the erratic thickness. Lee^1^ seemed to be very energetic and young first researcher who discussed the flow via the thin moving needle. He perceived that the thickness displacement and drag per-unit length moderate very gradually while increasingly thin needle, but ultimately turn into zero as the needle disappears. It is significant to note that thin moving needle movement disturbs the path of the free-stream in the flow. This observable fact is the key to determine the temperature and velocity profiles in the flow of experimental type research. The handling of the thin moving needle is a progressively more imperative feature in industries of engineering and medicine. For example, problems with blood flow, anemometer hot wire to calculate the velocity of the wind, coating and lubricating of wires and transportation. Narain and Uberoi^2^ expanded the work of Lee by taking the combination of free and forced convective flow with heat transfer from a thin moving needle. They presented the similarity solution as well as a series solution. Wang^3^ scrutinized the mixed convection flow from a heated tip through a thin needle. Their results indicated that the solution is unique for aiding flow; however, the dual solutions exist for the opposing flow. Multiple solutions of axisymmetric flow passing from a slim needle moving in the opposite or the same path to the free stream were obtained by the researcher Ishak et al.^4^. Ahmed et al.^5^ discussed the influences of assisting flow as well as opposing flow from a thin needle. The Keller-box is utilized to obtain the numerical solutions. They observed that the flow with the characteristics of heat transfer is considerably influenced by the size of the needle and mixed convective parameter. Entropy analysis of flow passing from a thin needle in a parallel stream with radiation influence was investigated by Afridi et al.^6^. Their results have shown that the entropy generation shrinks due to the needle size. Recently, many researchers utilized the concept of nanofluid regarding the geometry of the thin needle. For an instant, Hayat et al.^7^ scrutinized the erratic heat flux impact close to a stagnation-point through the phenomena of heat transfer applying water-based carbon nanoliquid. The results indicated that the velocity appreciably augments due to nanoparticle volume fraction. Soid et al.^8^ presented the stability analysis of MHD radiative flow containing nanofluid through a thin needle and obtained the multiple results using bvp4c solver and they observed that the presence of nanoparticle, the domains of the solution are smaller. The Tiwari–Das nanoliquid model with the characteristic of heat transfer through a thin needle with mixed convection has been developed to achieve the multiple outcomes by Salleh et al.^9^. They performed the stability analysis and found that the upper branch solution is stable, while the lower one is unstable. Salleh et al.^10^ scrutinized the heat source on flow comprising nanoliquid through a thin needle with chemical reaction. They explored that dual outcomes exist only when the thin needle travels against the flow direction. The exploration of a magnetic field containing menthol based Al-Cu hybrid nanoliquid through a moving needle submerged horizontally was investigated by Sulochana et al.^11^. They have shown that due to the size of the needle, the rate of heat transport augments. Tlili et al.^12^ examined the slip effect on 3D magneto flow comprising menthol based hybrid (Cuo–MgO) nanofluid across an erratic surface. They have examined that the combination of Cuo–MgO works as an excellent insulator. Kumar et al.^13^ examined the influence of thermal radiation on the time-dependent flow of Casson fluid over a curved exponential heated surface with erratic heat source/sink and Joule heating. The thermal transfer of the time-dependent thin-film flow containing Oldroyd-B ferroliquid suspended in water-based CoFe_2_O_4_ (cobalt ferrite) with a magnetic field was scrutinized by Tlili et al.^14^. They observed that the time-dependent parameter and Deborah number has the command to control the rate of heat transfer. Recently, Tlili et al.^15^ inspected the 3D magneto flow containing aluminum alloys amalgamated hybrid nanofluid through an irregular thickness sheet with a slip impact. It has been observed that the stimulus of the magnetic field is smaller on hybrid nanoliquid compared to nanoliquid.

Properties of thermo-physical particularly the thermal conductivity of regular liquids like glycol, water, oil of the engine can be enhanced if the tiny sized particles (1–100 nm) are merged into the regular liquids. These merged liquids are named as nanoliquid pioneered by Choi et al.^16^ in line to present the nanoliquids in the regular liquids in the form of engineered colloidal suspensions. Nanoliquids are composed of distinct materials like metals, ceramics, alloys, nanotube, semi-conductors, and composite elements. In the last two decades, nanoliquid has been utilized as a superior liquid in heat transfer, particularly in the production of chemical, transportation, solar collector, cooling of electronics, power generation, and industries in biomedical^17–19^. The small size of the nanomaterials guides to improved stability of a suspension, the capability to flow efficiently without the obstruction of the system, and provided the improved physical as well as thermal properties. Also, it has been shown that nanoparticles existence escorts to an augmentation up to 15% to 40% in the regular fluid thermal conductivity. Haq et al.^20^ argued the combination of the thermal as well as velocity slip on MHD flow containing the nanoparticles from a stretched sheet through radiation effect. They observed that the Lorentz force declines the velocity and uplift the temperature, while the slip effect declines the heat transfer. Hayat et al.^21^ utilized the condition of mass flux to discuss the heat absorption/generation impact on flow comprising nanofluid from a stretching sheet with nonlinear radiation. They found that that heat absorption/generation uplifts the temperature as well as the heat transfer rate. Rehman et al.^22^ utilized the new condition of mass flux on flow with the heat transfer involving revised second-grade nanoliquid through a stretched nonlinear sheet. They have explored that the concentration of nanofluid became weaker due to the Lewis number. The impact of bio-convection with nanoparticles in second-grade liquid through a thin film with gyrotatic microorganisms in the presence of the passive control condition was discussed by Khan^23^. He observed that the velocity depreciated due to the Rayleigh number. Ahmed et al.^24^ scrutinized the nonlinear radiation effect on the time-dependent thin-film flow comprising the Maxwell nanoliquid through a rotating disk with activation energy. The outcomes exposed that the temperature of nanofluid significantly augments owing to the impact of thermophoresis. Wakif et al.^25^ reported thermo migration of nano/tiny-sized particles through the various fluids motion. Their outcomes have shown that the drag force decreases due to thermophoresis. Riaz et al.^26^ deliberated the impact of slip on the peristaltic flow containing nano-sized particles through a curved channel in a porous medium. They also added the creeping flow owing to the small Reynolds number. Babazadeh et al.^27^ reported the modeling of migration of magneto nanoparticle inside a porous space with radiation effect and shape factor. They observed that the greater convection mode can be gained due to the buoyancy force. Majeed et al.^28^ explored the characteristics of heat transfer comprising the magneto ferrofluid with dipole impact by controlling the thermal and momentum boundary-layer region. They concluded that the temperature of nanofluid decelerates due to nanoparticle. Tarakaramu and Satya Narayana^29^ studied the influence of heat source on the magneto time-dependent flow of Eyring-Powell nanofluid through a moving surface with chemical reaction and radiation effects. Recently, Tlili et al.^30^ premeditated the time-dependent free convection flow through the mixture of engine oil based TC4/NiCr nanofluid from a revolving cone in a porous medium. The transmission of the heat transfer rate of hybrid nanofluid is moderately lesser than the regular fluid.

The scrutiny of non-Newtonian liquids in the current era has received immense interest due to their industrial and engineering intention. These liquids are indispensable, especially for the manufacturing of materials, production of a chemical and lubricants, mixtures of the polymer, etc. It is prominent that the investigation of non-Newtonian liquids and their properties contain numerous complexities due to the addition of rheological impacts in the constitutive leading equations. These supplementary physical and rheological quantities provide to augment the further complex and non-linear equations. Among different models, the Williamson liquid model is one of the signifying models. This model has an unambiguous benefit over other non-Newtonian models since it holds a minimum as well as maximum viscosities which give better outcomes for pseudoplastic liquids. Khan and Khan^31^ employed the HAM technique to get the series solution of Williamson liquid. The analysis of two-dimensional flow involving Williamson liquid from an exponential and linear stretching surface was deliberated by Nadeem et al.^32,33^. Hayat et al.^34^ explored MHD impact on time-dependent flow from a porous plate with fluid such as Williamson. They found that the velocity gradient decreases due to Williamson parameter. The effect of chemical reaction on the electrical conducting flow of Williamson nanoliquid from a stretched sheet immersed in the porous media with melting heat transfer was scrutinized by Krishnamurthy et al.^35^. They examined that the drag force and heat transfer are higher due to the presence of nanoparticle. The chemical reaction and radiation on Newtonian as well as non-Newtonian liquids near a stagnation-point from an incline cylindrical sheet in a dual stratified medium were evaluated by Rahman et al.^36^. They concluded that the heat transfer decreases due to thermophoresis and Brownian parameters. The time-dependent flow comprising Williamson fluid through a porous stretched sheet with MHD suspended nanoliquid was reviewed by Bibi et al.^37^. The radiation impact on the time-dependent MHD flow of Williamson fluid in the existence of nanoliquid through the radially stretched surface was scrutinized by Hashim et al.^38^. They scrutinized that the heat transfer declines with augmenting the magnetic field. They concluded that the velocity of nanofluid and temperature uplift due to Brownian motion. Sarojamma et al.^39^ inspected the homogenous-heterogeneous reaction along with erratic thermal conductivity involving non-Newtonian fluid through an uneven surface. They revealed that the velocity decreases due to the wall parameter. Tarakaramu and Satya Narayana^40^ investigated the Lorentz motion of a non-Newtonian fluid through a linear stretched surface with heat generation embedded in a porous medium. They found that heat generation and magnetic fields are responsible for greater heat transport in the fluid flow. Recently, Sandhya et al.^41^ explored the impacts of activation energy and second-order slip on magneto buoyancy flow through an exponential stretched surface with radiation effect.

The usage of convection heat transfer along with non-Newtinian nanofluids is crucial to design several kinds of thermal equipments. Thus, the main aim of the current problem is to scrutinize the effect of MHD involving titanium alloy nanoparticle with Williamson fluid past a thin needle. Also, the impact of nonlinear radiation is invoked. Tiwari–Das model is taken to simulate the flow problem. The dual complex nature of solutions are obtained which most of the researchers missed out. Also, the stability analysis is also performed. Moreover, the accumulation of Williamson liquid containing magnetite titanium alloy nanoparticle composes a more complex mixture compared to the host nanoliquid. This explores an incorporate a novel epoch for researchers to determine the heat transfer containing the titanium alloy nanoliquid characteristics. In addition, Impacts of the significant constraints elucidate in detail with the assistance of plots and tabular form.

## Mathematical formulation

**Figure 1:**
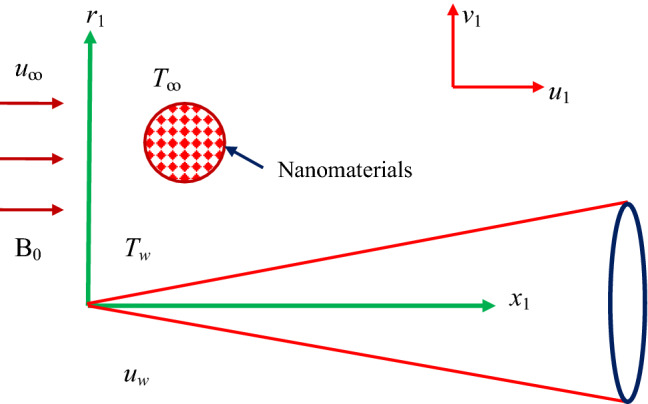
Geometry of the problem.

As mentioned in Fig. 1, the two-dimensional incompressible flow of titanium alloy nanoparticle in the presence of Williamson liquid through a thin needle is scrutinized. The coordinates are utilized in cylindrical form $\left( {x_{1} ,r_{1} } \right)$, where the coordinates $r_{1}$- and $x_{1}$- signifies radial and axial directions, respectively. Further, we incorporate the impacts of nonlinear radiation and magneto-hydrodynamics (MHD) on flow with characteristics of heat transfer. It is prominent that the needle is deemed as slim whilst the thickness of the needle doesn’t surpass that of the boundary layer flow (BLF) over it. Since the size of the needle is small, the pressure gradient is disregarded. Though, the impact of transverse-curvature is needed. The erratic magnetic field $B(x_{1} ) = B_{0} /x_{1}^{0.5}$ is inflicted in the path of the moving fluid while induced magnetic is unnoticed owing to the negligibly small Reynolds number. Also, it is perceived that $T_{\infty }$ and $T_{w}$ signify the ambient and constant temperature of a thin needle with $T_{w} > T_{\infty }$. Moreover, the needle travels through steady velocity by $u_{w}$ on the contrary or identical direction to the velocity of free-stream which is $u_{\infty }$. The Cauchy stress-tensor for the Williamson fluid is suggeted by Dapra et al.^42^ as

1 $$ {\text{S}}_{1} = - p{\rm I} + \tau_{1} , $$

2 $$ \tau_{1} = \left( {\mu_{\infty } + \frac{{\mu_{0} - \mu_{\infty } }}{{1 - \Gamma_{1} \dot{\gamma }_{1} }}} \right){\text{A}}_{{1}} , $$

Here ${\text{S}}_{1}$ is the extra stress-tensor, $\mu_{0}$ and $\mu_{\infty }$, respectively are limiting viscosities at zero and infinite shear rates, $p$ the pressure, ${\text{I}}$ the identity vector, $\Gamma_{1} ,{\text{A}}_{{1}}$ represent the time relaxation and the first tensor of Rivlin-Erickson, respectively and $\dot{\gamma }_{1}$ is presented in the following form

3 $$ \dot{\gamma }_{1} = \sqrt {\frac{1}{2}\pi } , $$

where $$\pi = trace\left( {{\text{A}}_{1}^{2} } \right).$$

Here we discussed the case in which $\Gamma_{1} \dot{\gamma }_{1} < 1$ and $\mu_{\infty }$. Therefor, $\tau_{1}$ can be simplified as

4 $$ \tau_{1} = \left( {\frac{{\mu_{0} }}{{1 - \Gamma_{1} \dot{\gamma }_{1} }}} \right){\text{A}}_{{1}} , $$

After employing the bionomial expansion, one gets

5 $$ \tau_{1} = \mu_{0} \left( {1 + \Gamma_{1} \dot{\gamma }_{1} } \right){\text{A}}_{{1}} . $$

The expressions of governing flow with these postulations are^4,6,38^

6 $$ \frac{\partial }{{\partial r_{1} }}\left( {r_{1} v_{1} } \right) + \frac{\partial }{{\partial x_{1} }}\left( {r_{1} u_{1} } \right) = 0 $$

7 $$ \frac{{\mu_{nanof} }}{{\rho_{nanof} }}\left[ {\frac{1}{{r_{1} }}\frac{\partial }{{\partial r_{1} }}\left( {r_{1} \frac{{\partial u_{1} }}{{\partial r_{1} }}} \right) + \sqrt 2 \Gamma_{1} \frac{{\partial u_{1} }}{{\partial r_{1} }}\frac{{\partial^{2} u_{1} }}{{\partial r_{1}^{2} }} + \frac{{\Gamma_{1} }}{{\sqrt 2 r_{1} }}\left( {\frac{{\partial u_{1} }}{{\partial r_{1} }}} \right)^{2} } \right] - \frac{{\sigma_{nanof} B^{2} (x_{1} )}}{{\rho_{nanof} }}u_{1} = v_{1} \frac{{\partial u_{1} }}{{\partial r_{1} }} + u_{1} \frac{{\partial u_{1} }}{{\partial x_{1} }} $$

8 $$ \alpha_{nanof} \frac{1}{{r_{1} }}\frac{\partial }{{\partial r_{1} }}\left( {r_{1} \frac{{\partial T_{1} }}{{\partial r_{1} }}} \right) + \frac{1}{{\left( {\rho c_{p} } \right)_{nanof} }}\left( {\frac{{16\sigma^{*} T_{1}^{3} }}{{3k^{*} }}\frac{{\partial^{2} T_{1} }}{{\partial r_{1}^{2} }} + \frac{{16\sigma^{*} 3T_{1}^{2} }}{{3k^{*} }}\left( {\frac{{\partial T_{1} }}{{\partial r_{1} }}} \right)^{2} } \right) = u_{1} \frac{{\partial T_{1} }}{{\partial x_{1} }} + v_{1} \frac{{\partial T_{1} }}{{\partial r_{1} }}, $$

The substantial boundary-conditions are

9 $$ \begin{aligned} & T_{1} = T_{w} ,v_{1} = 0,u_{1} = u_{w} {\text{ at }}r_{1} = R(x_{1} ), \\ & T_{1} \to T_{\infty } ,u_{1} \to u_{\infty } {\text{ as }}r_{1} \to \infty . \\ \end{aligned} $$

Here, $\left( {v_{1} ,u_{1} } \right)$ signify respectively the radial and axial velocity components, $\left( {x_{1} ,r_{1} } \right)$ the coordinates in cylindrical form, $\rho_{nanof}$ density of nanoliquid, $\mu_{nanof}$ viscosity of nanoliquid, $\left( {\rho c_{p} } \right)_{nanof}$ heat capacity of nanoliquid, $\sigma_{nanof}$ electric conductivity of nanofluids, $\sigma^{*}$ Stefan Boltzmann constant, $k^{*}$ coefficient of mean absorption. The nanoliquid quantities are described as^7–9^

10 $$ \begin{aligned} \left( {\rho c_{p} } \right)_{nanof} & = \phi \left( {\rho c_{p} } \right)_{s} + \left( {1 - \phi } \right)\left( {\rho c_{p} } \right)_{f} ,\mu_{nanof} = \left( {1 - \phi } \right)^{ - 2.5} \mu_{f} , \\ \alpha_{nanof} & = k_{nanof} \left\{ {\left( {\rho c_{p} } \right)_{nanof} } \right\}^{ - 1} , \\ k_{nanof} /k_{f} & = \left\{ {\left( {k_{s} + 2k_{f} } \right) + \phi \left( {k_{f} - k_{s} } \right)} \right\}^{ - 2.5} \left\{ {\left( {k_{s} + 2k_{f} } \right) - 2\phi \left( {k_{f} - k_{s} } \right)} \right\}, \\ \rho_{nanof} & = \phi \rho_{s} + \left( {1 - \phi } \right)\rho_{f} ,\sigma_{nanof} /\sigma_{f} = \left[ {1 + \left\{ {\left( {\sigma_{s} /\sigma_{f} + 2} \right) - \left( {\sigma_{s} /\sigma_{f} - 1} \right)\phi } \right\}^{ - 2.5} \left\{ {3\left( {\sigma_{s} /\sigma_{f} - 1} \right)\phi } \right\}} \right]. \\ \end{aligned} $$

where $\phi$ called the volume fraction of nanoliquid, $\left( {\rho_{s} ,\rho_{f} } \right)$ signify the densities of nanoliquid and regular liquid, $\mu_{f}$ the viscosity of a regular liquid, $\left( {k_{s} ,k_{f} } \right)$ portrays the thermal conductivities of nanoliquid and regular liquid, respectively. 

We want to ease the computation of the current model owing to imply the following similarity variables as:

11 $$ \psi_{1} = \nu_{f} x_{1} F\left( \eta \right), \, \theta \left( \eta \right) = \frac{{\left( {T_{1} - T_{\infty } } \right)}}{{\left( {T_{w} - T_{\infty } } \right)}}, \, \eta = \frac{{Ur_{1}^{2} }}{{\nu_{f} x_{1} }}. $$

Thus, in the aforementioned Eq. (11), the description of symbols is followed as $\nu_{f}$ the kinetic viscosity, $U$ the merged velocity classified as $U = u_{\infty } + u_{w}$, and $\psi_{1}$ the stream function is described as $v_{1} = - \left( {\frac{1}{{r_{1} }}} \right)\frac{{\partial \psi_{1} }}{{\partial x_{1} }}$ and $u_{1} = \left( {\frac{1}{{r_{1} }}} \right)\frac{{\partial \psi_{1} }}{{\partial r_{1} }}$. Substituting $\eta = c$ in the Eq. (11), which portrays the size and as well as the shape of the thin moving needle $r_{1} = R(x_{1} )$ through its surface, which is specified via $R(x_{1} ) = \left( {\nu_{f} cx_{1} U^{ - 0.5} } \right)^{0.5}$.

Utilizing (11), the governing Eqs. (7)–(9) are converted as

12 $$ \begin{aligned} & F^{\prime\prime\prime} \left( {2\eta + 8\eta WeF^{\prime\prime}} \right) + 2F^{\prime\prime} + 6We\left( {F^{\prime\prime}} \right)^{2} + \left( {1 - \phi } \right)^{2.5} \left( {1 - \phi + \phi \frac{{\rho_{s} }}{{\rho_{f} }}} \right)FF^{\prime\prime} \\ & \quad - \left( {1 - \phi } \right)^{2.5} \frac{{\sigma_{nanof} }}{{\sigma_{f} }}MF^{\prime} = 0 \\ \end{aligned} $$

13 $$ \begin{aligned} & \frac{{k_{nanof} }}{{k_{f} }}\left( {\eta \theta ^{\prime\prime} + \theta ^{\prime}} \right) + \frac{\Pr }{2}\left( {\left( {1 - \phi } \right) + \phi \frac{{\left( {\rho c_{p} } \right)_{s} }}{{\left( {\rho c_{p} } \right)_{f} }}} \right)F\theta ^{\prime} \\ & \quad + \frac{4}{{3R_{d} }}\left( {\theta \left( {\theta_{r} - 1} \right) + 1} \right)^{2} \left[ {\left( {\theta \left( {\theta_{r} - 1} \right) + 1} \right)\left( {\eta \theta ^{\prime\prime} + \frac{\theta ^{\prime}}{2}} \right) + 3\theta ^{{\prime}{2}} \left( {\theta_{r} - 1} \right)\eta } \right] = 0 \\ \end{aligned} $$

14 $$ \begin{aligned} & \theta (c) = 1{,}F(c) = 0.5\lambda c,F^{\prime}(c) = 0.5\lambda , \\ & \theta (\eta ) \to 0,F^{\prime}\left( \eta \right) \to 0.5\left( {1 - \lambda } \right){\text{ as }}\eta \to \infty . \\ \end{aligned} $$

Here, the dimensionless variables are Prandtl number, magnetic, Weissenberg, local Reynolds numbers, radiation, velocity ratio and temperature ratio parameters, respectively. These parameters are described as

15 $$ \left\{ \begin{gathered} \Pr = \frac{{\nu_{f} }}{{\alpha_{f} }},M = \frac{{\sigma_{f} B_{0}^{2} }}{{2U\rho_{f} }},We = \frac{{\sqrt 2 \Gamma_{1} U^{2} r_{1} {\text{Re}}_{{x_{1} }} }}{{\nu_{f} x_{1} {\text{Re}}_{{r_{1} }} }},{\text{Re}}_{{x_{1} }} = \frac{{Ux_{1} }}{{\nu_{f} }},{\text{Re}}_{{r_{1} }} = \frac{{Ur_{1} }}{{\nu_{f} }},R_{d} = \frac{{k_{f} k^{*} }}{{4T_{\infty }^{3} \sigma^{*} }},\lambda = \frac{{u_{w} }}{U}, \hfill \\ \theta_{r} = \frac{{T_{w} }}{{T_{\infty } }}. \hfill \\ \end{gathered} \right. $$

It is important to mention that the domain of the velocity ratio parameter is set to $\left( {0 < \lambda < 1} \right)$ which specifies the fluid and needle progress in an identical direction. If $\lambda > 1$, then the needle is going near a positive $x_{1} -$ direction, whereas the free-stream shifts in the way of a negative $x_{1} -$ direction. Here, we consider only the case $\lambda \le 1$.

Mathematically, the temperature gradient and velocity gradient (heat transfer rate and the surface drag force) are identified as

16 $$ \left. \begin{gathered} Nu_{{x_{1} }} = \frac{{x_{1} q_{w} }}{{k_{f} (T_{w} - T_{\infty } )}}, \hfill \\ C_{f} = \frac{{\tau_{w} }}{{U^{2} \rho_{f} }}, \hfill \\ \end{gathered} \right\} $$

where $q_{w}$ and $\tau_{w}$ are recognized as the heat-flux and the shear stress respectively, which are specified as

17 $$ \left. \begin{gathered} q_{w} = \left. {\left( { - k_{nanof} \left( {\frac{{\partial T_{1} }}{{\partial r_{1} }}} \right) - \frac{{16\sigma^{ * } T_{1}^{3} }}{{3k^{ * } }}\left( {\frac{{\partial T_{1} }}{{\partial r_{1} }}} \right)} \right)} \right|_{{_{{r_{1} = c}} }} , \hfill \\ \tau_{w} = \mu_{nanof} \left( {\frac{{\partial u_{1} }}{{\partial r_{1} }}} \right)_{{r_{1} = c}} , \hfill \\ \end{gathered} \right\} $$

Utilizing (11), we have

18 $$ \left. {\begin{array}{*{20}l} {{\text{Re}}_{{x_{1} }}^{ - 1/2} Nu_{{x_{1} }} = - 2\sqrt c \theta ^{\prime}(c)\left( {\frac{{k_{nanof} }}{{k_{f} }} + \frac{4}{{3R_{d} }}\left( {\left( {\theta_{r} - 1} \right)\theta \left( c \right) + 1} \right)^{3} } \right),} \hfill \\ {{\text{Re}}_{{x_{1} }}^{1/2} C_{f} = \frac{{\mu_{nanof} }}{{\mu_{f} }}\frac{4\sqrt c F^{\prime\prime}(c)}{{(1 - \phi )^{2.5} }}.} \hfill \\ \end{array} } \right\} $$

## Stability analysis of the solution

The results numerically confirm that there occurs more than one solution called multiple (the lower branch (LB) and the upper branch (UB)) solutions and this idea is scrutinized in the Weidman et al.^43^, where they were exercising the stability analysis and established that which result is unstable and which one is stable. The key point to be noticed of this analysis is presented to obtain that which result provided the best path to the fluid flow mean physically realizable or stable result. For this purpose, more than one solution for various models has been investigated by Sharma et al.^44^, Yasin et al.^45^, Mansur et al.^46^, Roşca et al.^47^.

We check the analysis through taking all the governing equations (from(7, 8) in the unsteady form except the continuity equation it will remain unchanged as follow:

19 $$ \begin{gathered} \rho_{nanof} \left( {\frac{{\partial u_{1} }}{\partial t} + u_{1} \frac{{\partial u_{1} }}{{\partial x_{1} }} + v_{1} \frac{{\partial u_{1} }}{{\partial r_{1} }}} \right) = \mu_{nanof} \left[ {\frac{1}{{r_{1} }}\frac{\partial }{{\partial r_{1} }}\left( {r_{1} \frac{{\partial u_{1} }}{{\partial r_{1} }}} \right) + \sqrt 2 \Gamma_{1} \frac{{\partial u_{1} }}{{\partial r_{1} }}\frac{{\partial^{2} u_{1} }}{{\partial r_{1}^{2} }} + \frac{{\Gamma_{1} }}{{\sqrt 2 r_{1} }}\left( {\frac{{\partial u_{1} }}{{\partial r_{1} }}} \right)^{2} } \right] - \hfill \\ \sigma_{nanof} B^{2} (x_{1} )u_{1} , \hfill \\ \end{gathered} $$

20 $$ \left( {\rho c_{p} } \right)_{nanof} \left( {\frac{{\partial T_{1} }}{\partial t} + u_{1} \frac{{\partial T_{1} }}{{\partial x_{1} }} + v_{1} \frac{{\partial T_{1} }}{{\partial r_{1} }} - \alpha_{nanof} \frac{1}{{r_{1} }}\frac{\partial }{{\partial r_{1} }}\left( {r_{1} \frac{{\partial T_{1} }}{{\partial r_{1} }}} \right)} \right) = \left( {\frac{{16\sigma^{*} T_{1}^{3} }}{{3k^{*} }}\frac{{\partial^{2} T_{1} }}{{\partial r_{1}^{2} }} + \frac{{16\sigma^{*} 3T_{1}^{2} }}{{3k^{*} }}\left( {\frac{{\partial T_{1} }}{{\partial r_{1} }}} \right)^{2} } \right), $$

The new dimensionless time variable is defined in the form $\tau = 2Ut/x_{1}$ , where $t$ stands for time. The new similarity transformations were followed as:

21 $$ \psi_{1} = \nu_{f} x_{1} F\left( {\eta ,\tau } \right),\,\,\theta \left( {\eta ,\tau } \right) = \frac{{T_{1} - T_{\infty } }}{{T_{w} - T_{\infty } }},\eta = \frac{{Ur_{1}^{2} }}{{\nu_{f} x_{1} }},\tau = \frac{2Ut}{{x_{1} }}. $$

By exercising the similarity transformations (21) into Eqs. (19) and (20), we get

22 $$ \begin{aligned} & 2\frac{{\partial^{2} F}}{{\partial \eta^{2} }} + 2\eta \frac{{\partial^{3} F}}{{\partial \eta^{3} }} + We\left( {6\left( {\frac{{\partial^{2} F}}{{\partial \eta^{2} }}} \right)^{2} + 8\eta \frac{{\partial^{2} F}}{{\partial \eta^{2} }}\frac{{\partial^{3} F}}{{\partial \eta^{3} }}} \right) - \left( {1 - \phi } \right)^{2.5} \frac{{\sigma_{nanof} }}{{\sigma_{f} }}M\frac{\partial F}{{\partial \eta }} \\& \quad + \left( {1 - \phi + \phi \frac{{\rho_{s} }}{{\rho_{f} }}} \right)\left( {\left( {1 - \phi } \right)^{2.5} \tau \frac{{\partial^{2} F}}{\partial \eta \partial \tau }\frac{\partial F}{{\partial \eta }} + \left( {1 - \phi } \right)^{2.5} F\frac{{\partial^{2} F}}{{\partial \eta^{2} }} - \left( {1 - \phi } \right)^{2.5} \frac{{\partial^{2} F}}{\partial \eta \partial \tau }} \right) = 0, \\ \end{aligned} $$

23 $$ \begin{aligned} & \frac{{k_{nanof} }}{{k_{f} }}\left( {\eta \frac{{\partial^{2} \theta }}{{\partial \eta^{2} }} + \frac{\partial \theta }{{\partial \eta }}} \right) + \frac{4}{{3R_{d} }}\left( {\theta \left( {\theta_{r} - 1} \right) + 1} \right)^{2} \left( {\left( {\theta \left( {\theta_{r} - 1} \right) + 1} \right)\left( {\eta \frac{{\partial^{2} \theta }}{{\partial \eta^{2} }} + \frac{1}{2}\frac{\partial \theta }{{\partial \eta }}} \right) + 3\left( {\frac{\partial \theta }{{\partial \eta }}} \right)^{2} \left( {\theta_{r} - 1} \right)\eta } \right) \\& + \quad \frac{\Pr }{2}\left( {\left( {1 - \phi } \right) + \phi \frac{{\left( {\rho c_{p} } \right)_{s} }}{{\left( {\rho c_{p} } \right)_{f} }}} \right)\left( {F\frac{\partial \theta }{{\partial \eta }} - \frac{\partial \theta }{{\partial \tau }} + \tau \frac{\partial F}{{\partial \eta }}\frac{\partial \theta }{{\partial \tau }}} \right) = 0,s, \\ \end{aligned} $$

along the corresponding condition are

24 $$ \begin{aligned} & \theta \left( {c,\tau } \right) - 1 = 0,F\left( {c,\tau } \right) - 0.5\lambda c = 0,\frac{{\partial F\left( {c,\tau } \right)}}{\partial \eta } - 0.5\lambda = 0, \\ & \theta \left( {\infty ,\tau } \right) \to 0,\frac{{\partial F\left( {\infty ,\tau } \right)}}{\partial \eta } = 0.5\left( {1 - \lambda } \right). \\ \end{aligned} $$

Then, we consider that^27,32^

25 $$ \begin{aligned} F\left( {\eta ,\tau } \right) - e^{ - \xi \tau } H\left( \eta \right) & = f_{0} (\eta ) \\ \theta (\eta ,\tau ) - e^{ - \xi \tau } G\left( \eta \right) & = \theta_{0} (\eta ). \\ \end{aligned} $$

To investigate the temporal stability to specify the answers $F = f_{0} (\eta )$ and $\theta = \theta_{0} (\eta )$ which joined the two-point problem (22) and (23). However, $\xi$ stand for the unknown eigenvalue parameter and as well as the functions $G\left( \eta \right)$ and $H\left( \eta \right)$ are fairly small as compared to $\theta_{0} (\eta )$ and $f_{0} (\eta )$. Plugging Eq. (25) into Eqs. (22) and (23) yields the following problem of linearized eigenvalue.

26 $$ \begin{aligned} & 2H_{0} ^{\prime\prime} + 2H_{0} ^{\prime\prime\prime} + 4We\left( {2\eta f_{0} ^{\prime\prime}H_{0} ^{\prime\prime} + 3f_{0} ^{\prime\prime}H_{0} ^{\prime\prime\prime} + 2\eta f_{0} ^{\prime\prime\prime} H_{0} ^{\prime\prime}} \right) - \frac{{\sigma_{nanof} }}{{\sigma_{f} }}\left( {1 - \phi } \right)^{2.5} MH_{0} ^{\prime} \\ & \quad + \left( {1 - \phi + \phi \frac{{\rho_{s} }}{{\rho_{f} }}} \right)\left( {\left( {1 - \phi } \right)^{2.5} f_{0} ^{\prime\prime}H_{0} + \left( {1 - \phi } \right)^{2.5} f_{0} H_{0} ^{\prime\prime} - \left( {1 - \phi } \right)^{2.5} \xi H_{0} ^{\prime}} \right) = 0, \\ \end{aligned} $$

27 $$ \begin{aligned} & \frac{{k_{nanof} }}{{k_{f} }}\left( {G_{0} ^{\prime} + \eta G_{0} ^{\prime\prime}} \right) + \frac{\Pr }{2}\left( {\left( {1 - \phi } \right) + \phi \frac{{\left( {\rho c_{p} } \right)_{s} }}{{\left( {\rho c_{p} } \right)_{f} }}} \right)\left( {f_{0} G_{0} ^{\prime} + H_{0} \theta_{0} ^{\prime} + \varsigma G_{0} } \right) + 2\left( {\left( {\theta_{r} - 1} \right)\theta_{0} + 1} \right)G_{0} \left( {\theta_{0} ^{\prime}} \right)^{2} \\ & \quad + 2\left( {\left( {\theta_{r} - 1} \right)\theta_{0} + 1} \right)^{2} \theta_{0} ^{\prime}G_{0} ^{\prime} + \frac{12}{{3R_{d} }}\left( {\theta_{r} - 1} \right)G_{0} \left( {\left( {\theta_{r} - 1} \right)\theta_{0} + 1} \right)^{2} \left( {\eta \theta_{0} ^{\prime\prime} + \frac{{\theta_{0} ^{\prime}}}{2}} \right) \\ & \quad + \frac{4}{{3R_{d} }}\left( {\left( {\theta_{r} - 1} \right)\theta_{0} + 1} \right)^{3} \left( {\eta G_{0} ^{\prime\prime} + \frac{{G_{0} ^{\prime}}}{2}} \right) = 0, \\ \end{aligned} $$

and the subjected conditions (18) are follow as

28 $$ \begin{aligned} & H_{0} ^{\prime}\left( c \right) = 0,H_{0} \left( c \right) = 0,G_{0} \left( c \right) = 0, \\ & H_{0} ^{\prime}\left( \infty \right) \to 0,G_{0} \left( \infty \right) \to 0. \\ \end{aligned} $$

Working out the Eqs. (26)-(28) numerically, we acquire the infinite eigenvalues $\left( {\xi_{1} < \xi_{2} < \xi_{3} < \xi_{4} \ldots } \right)$. The flow is said to be physically realizable (stable) if $\xi > 0$ while it is not physically realizable (unstable) for the choice of $\xi < 0$. The range for the eigenvalue is specified by Harris et al.^48^ where they have relaxing conditions on $G_{0} \left( \eta \right)$ and $H_{0} \left( \eta \right)$. In this recent work, we take $H_{0} ^{\prime}\left( \eta \right) \to 0$ as $\eta \to \infty$ and then tackled the Eqs. (26) and (27) together with (27) and along with once more new condition which is $H_{0} ^{\prime}\left( 0 \right) = 1.$

## Physical explanation

**Figure 2:**
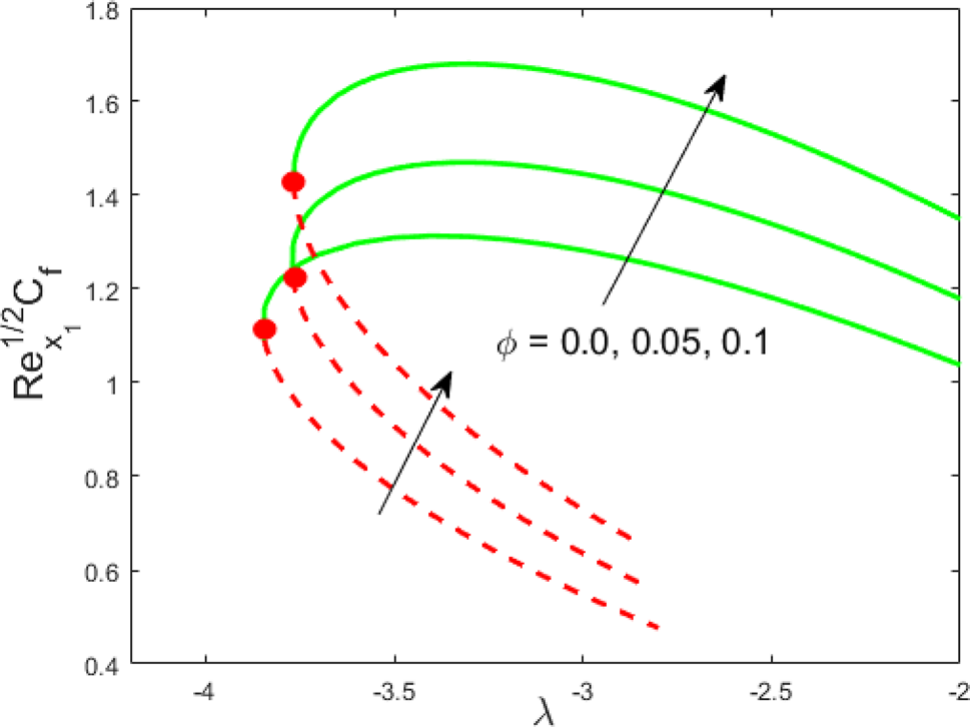
Influence of $\phi$ on ${\text{Re}}_{{x_{1} }}^{1/2} C_{f}$.

**Figure 3:**
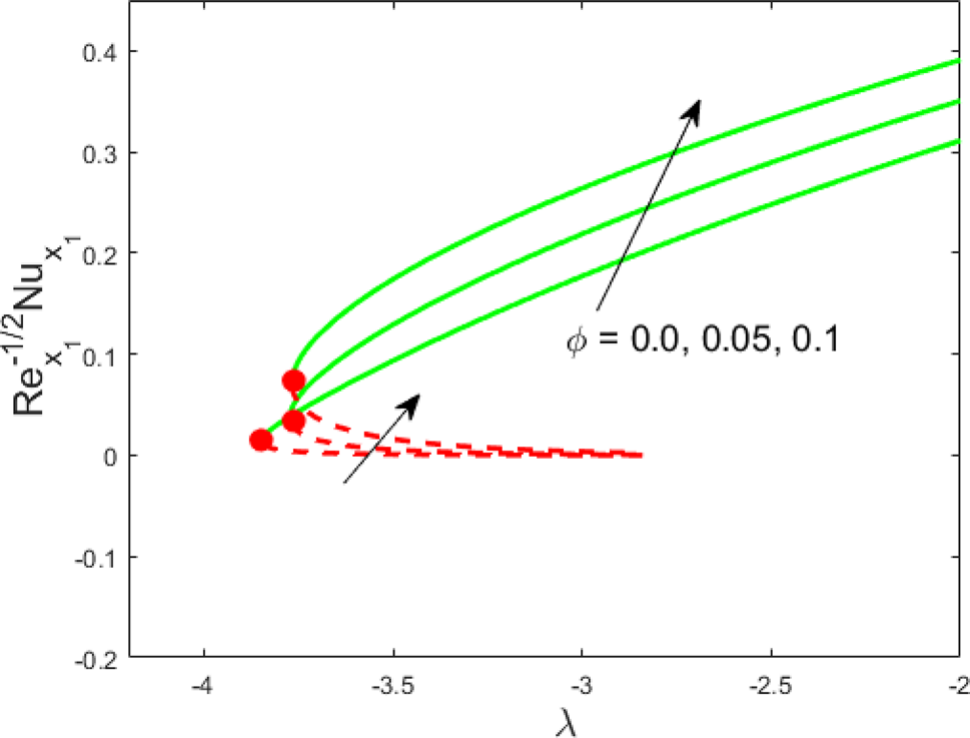
Influence of $\phi$ on ${\text{Re}}_{{x_{1} }}^{ - 1/2} Nu_{{x_{1} }}$.

**Figure 4:**
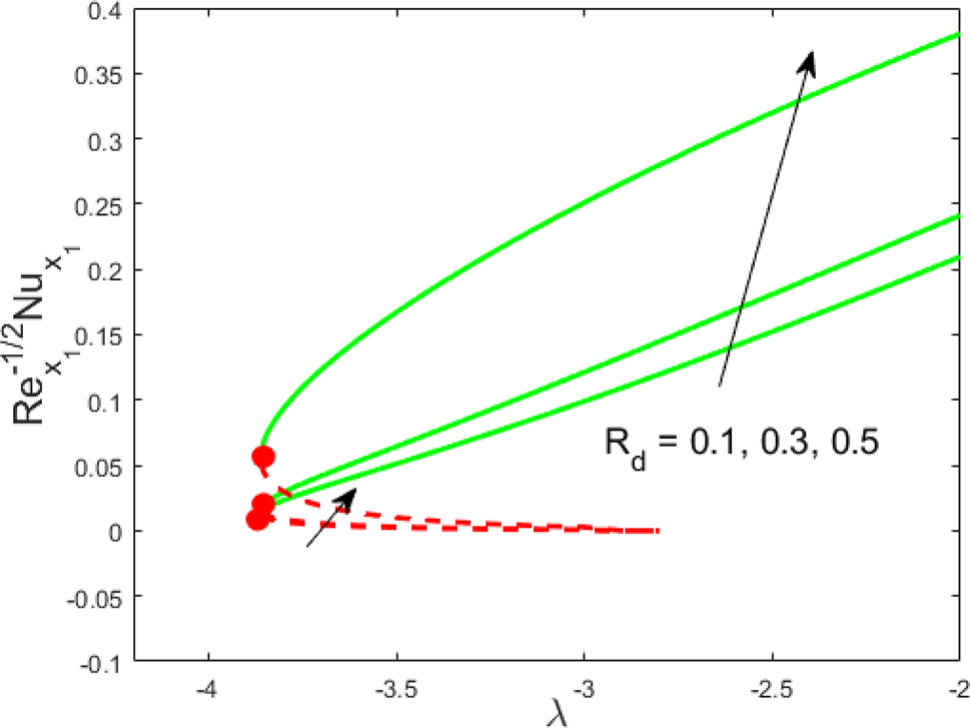
Influence of $R_{d}$ on ${\text{Re}}_{{x_{1} }}^{ - 1/2} Nu_{{x_{1} }}$.

**Figure 5:**
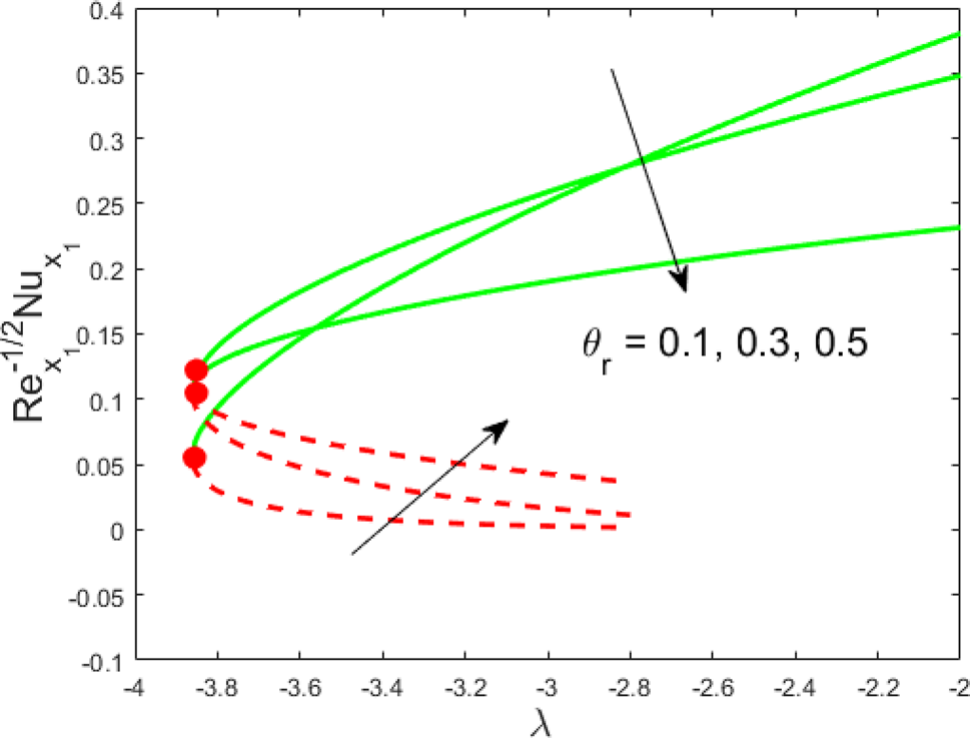
Influence of $\theta_{r}$ on ${\text{Re}}_{{x_{1} }}^{ - 1/2} Nu_{{x_{1} }}$.

**Figure 6:**
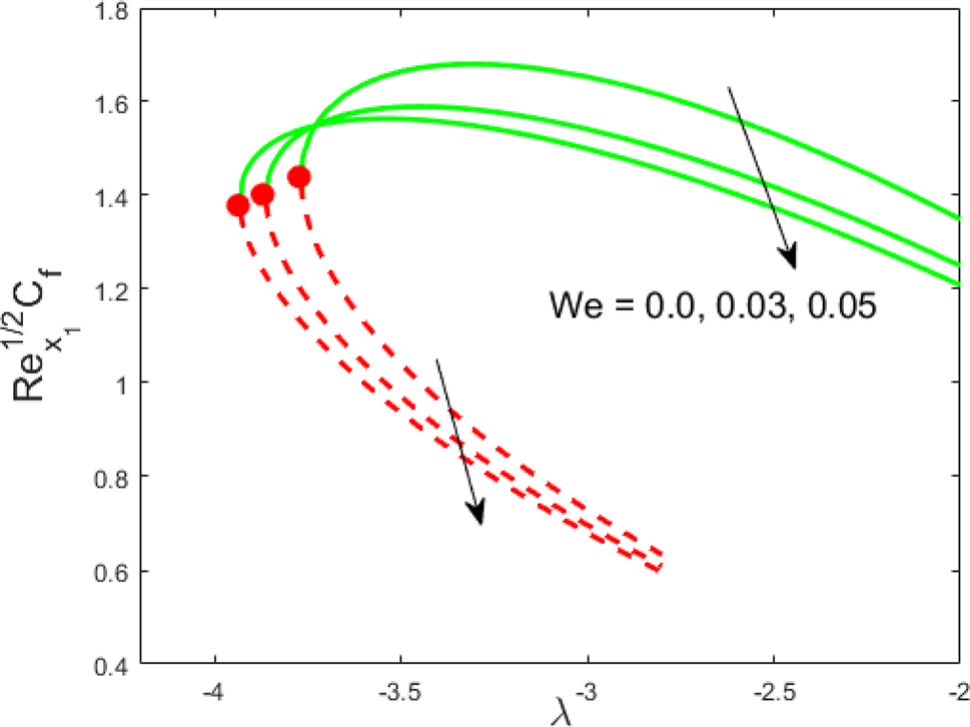
Influence of $We$ on ${\text{Re}}_{{x_{1} }}^{1/2} C_{f}$.

**Figure 7:**
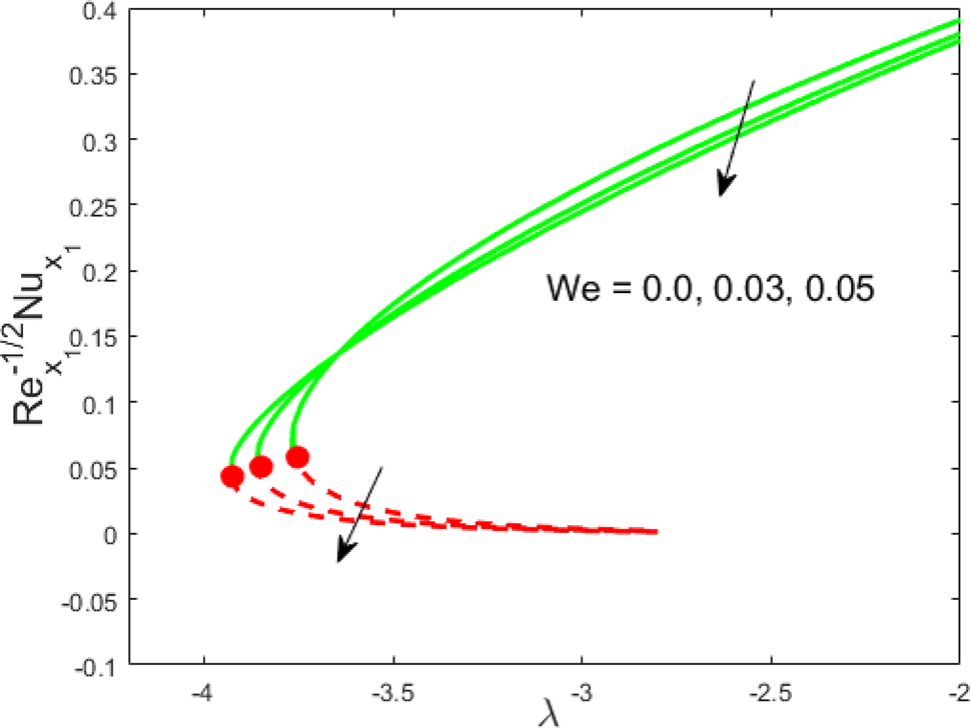
Influence of $We$ on ${\text{Re}}_{{x_{1} }}^{ - 1/2} Nu_{{x_{1} }}$.

**Figure 8:**
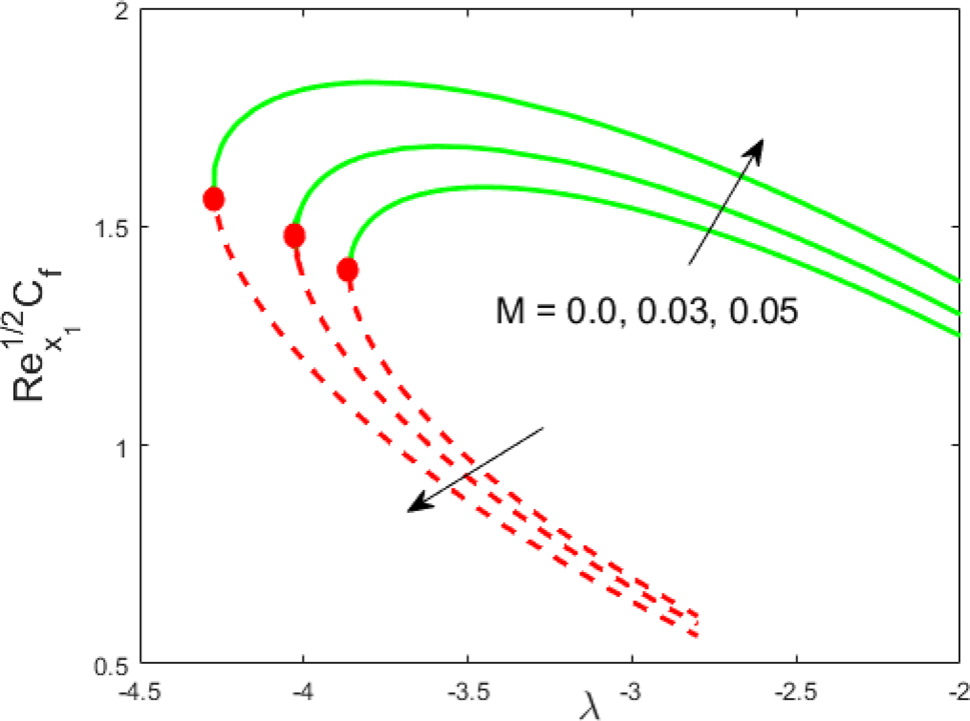
Influence of $M$ on ${\text{Re}}_{{x_{1} }}^{1/2} C_{f}$.

**Figure 9:**
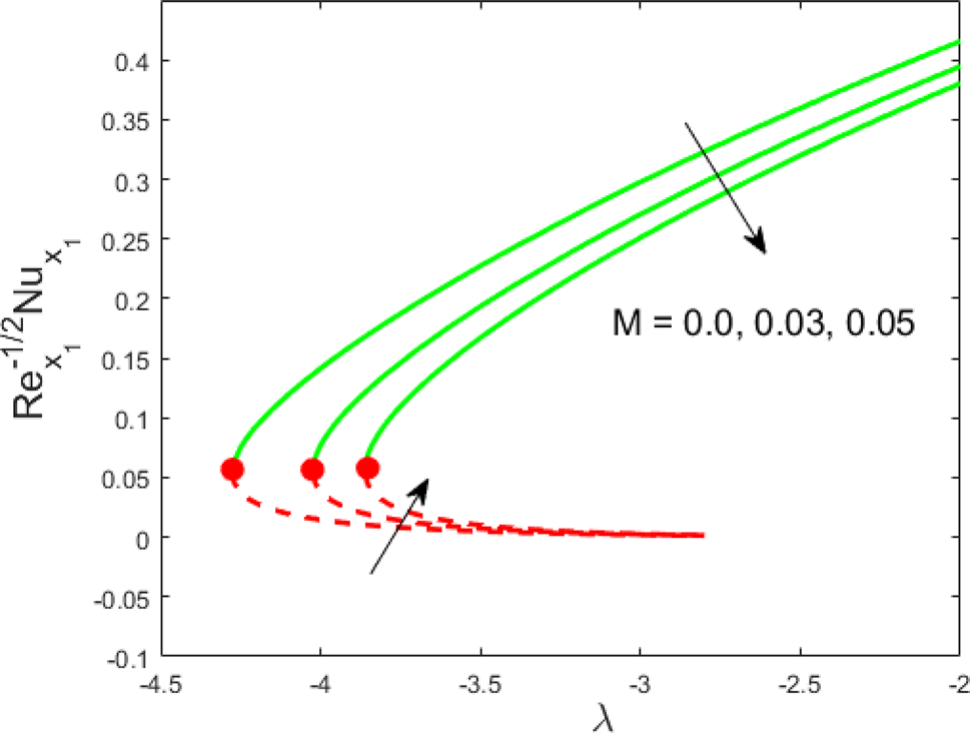
Influence of $M$ on ${\text{Re}}_{{x_{1} }}^{ - 1/2} Nu_{{x_{1} }}$.

**Figure 10:**
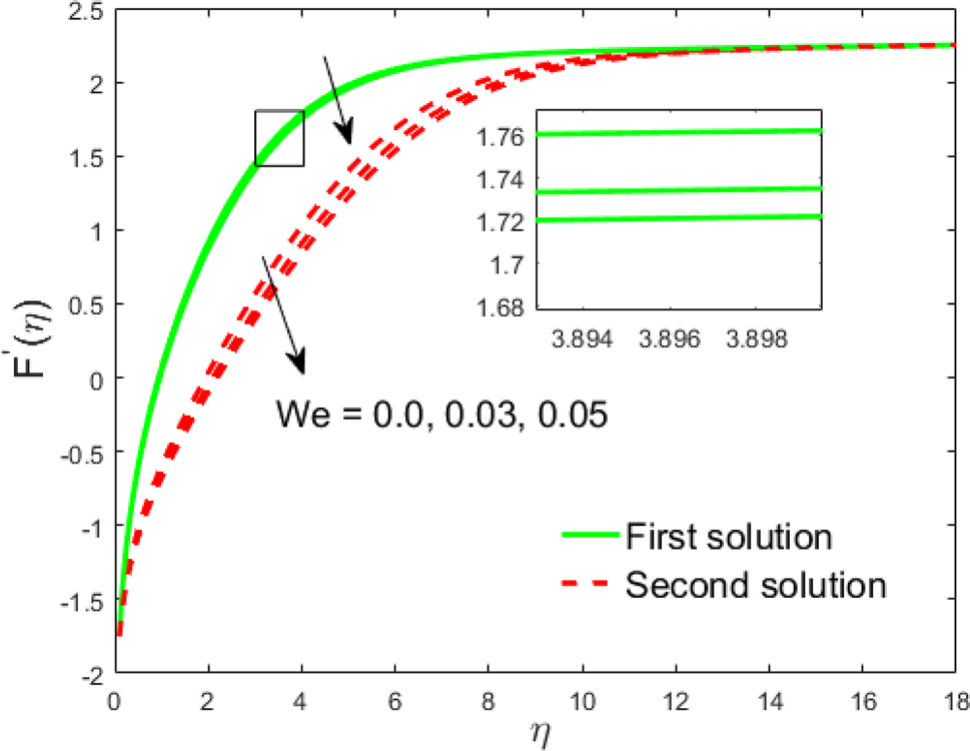
Influence of $We$ on $F^{\prime}\left( \eta \right)$.

**Figure 11:**
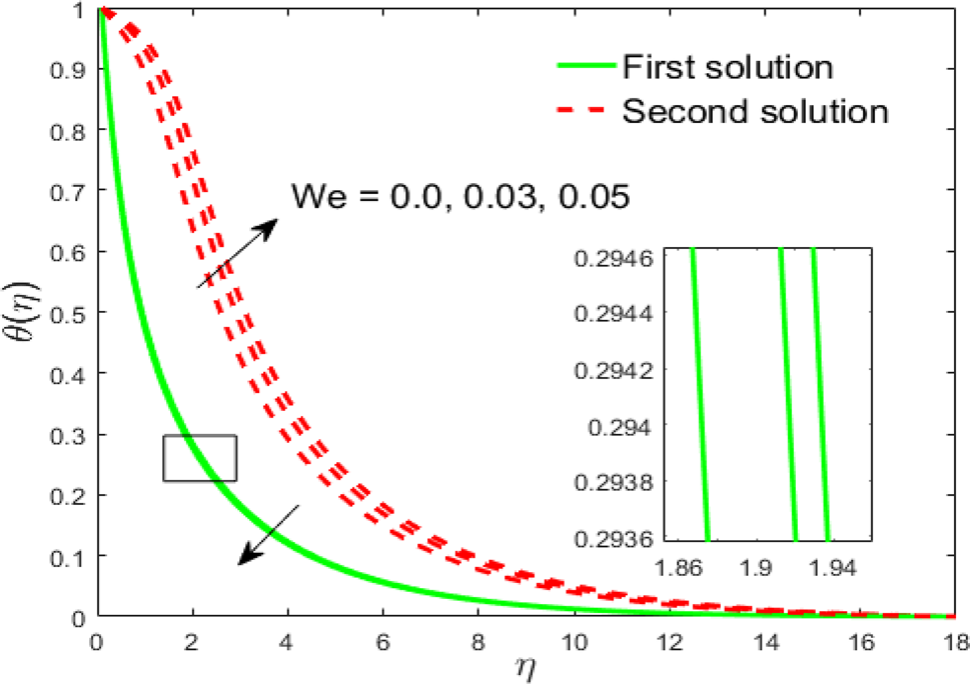
Influence of $We$ on $\theta \left( \eta \right)$.

**Figure 12:**
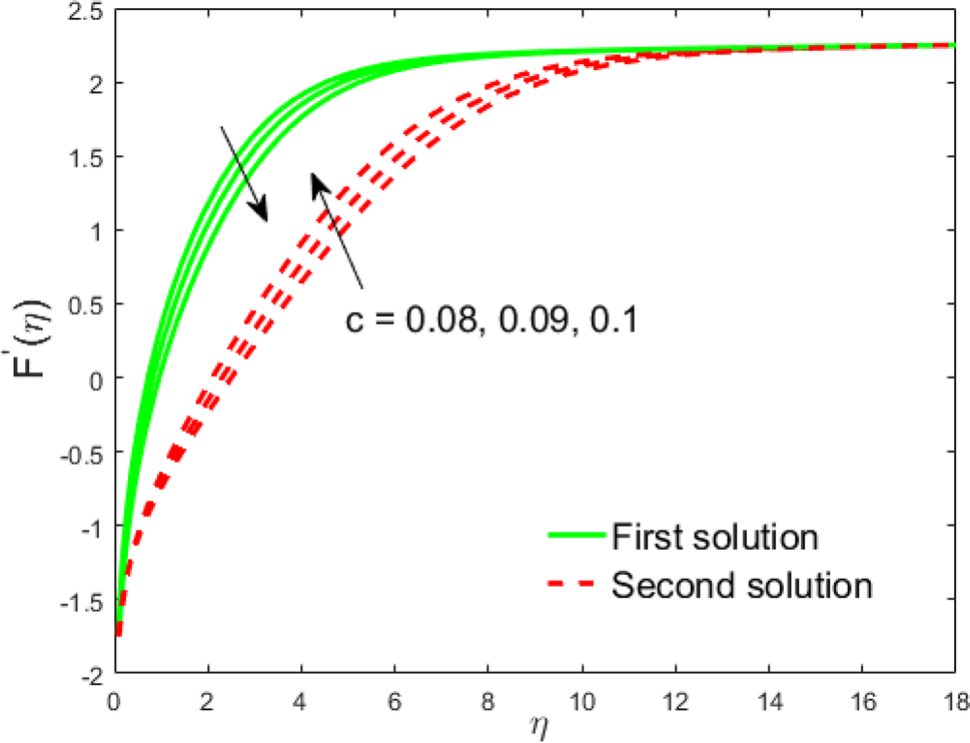
Influence of $c$ on $F^{\prime}\left( \eta \right)$.

**Figure 13:**
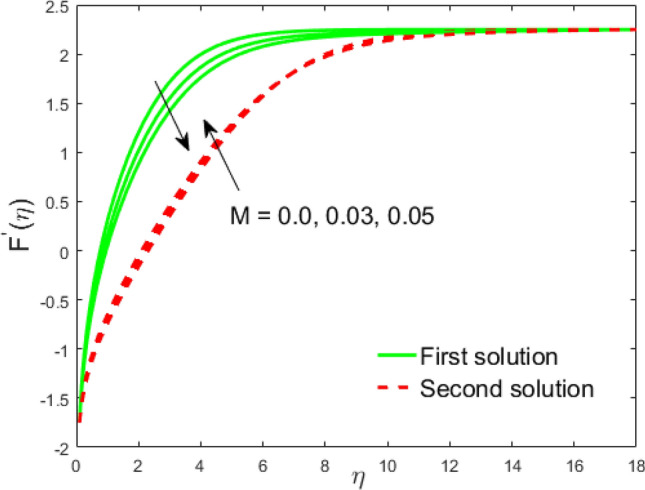
Influence of $M$ on $F^{\prime}\left( \eta \right)$.

**Figure 14:**
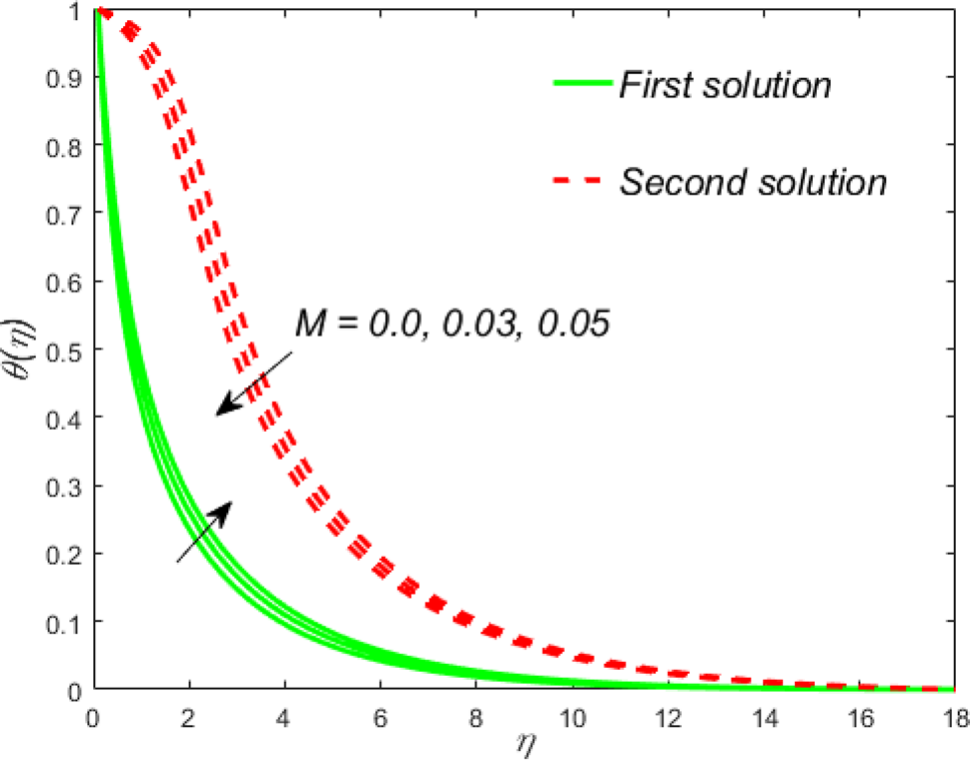
Influence of $M$ on $\theta \left( \eta \right)$.

**Figure 15:**
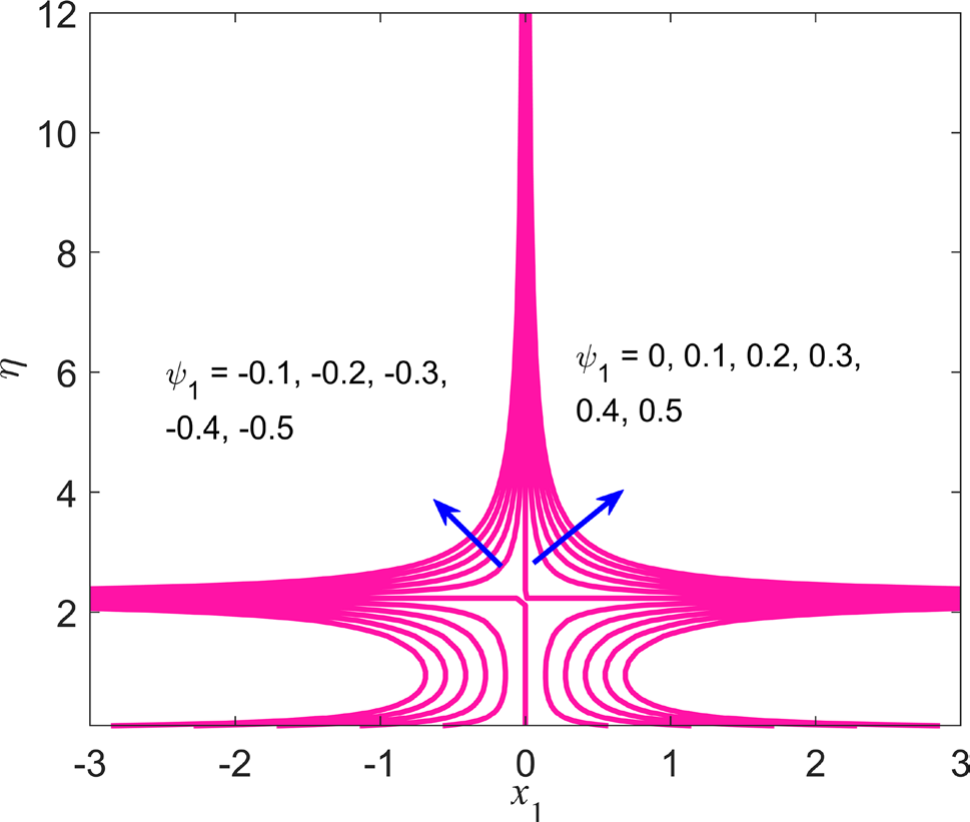
Streamlines pattern for nanofluid.

The transmuted nonlinear ODEs (12) and (13) along with boundary restriction (14) are tackled numerically with the help of bvp4c solver via the formula of 3-stage Lobatto IIIa. The characteristics and features of different pertinent variables appeared in the problem on the profiles of liquid velocity, temperature, Nusselt number, and the skin friction are portrayed in Figs. 2, 3, 4, 5, 6, 7, 8, 9, 10, 11, 12, 13, 14 and 15. The values appeared in the problem during the computation are considered fixed as $\theta_{r} = R_{d} = c = 0.1$, $We = 0.05$, $M = 0.1$, $\lambda = - 3.5$, $\phi = 0.1$. Table 1 depicts the significance of the physical and thermal properties of titanium alloy nanoparticle. A comparison of our results with available outcomes is made in Table 2 to validate our problem and discovered in a tremendous closeness. Also, Table 3 is constructed for comparision between two techniques and they are in an excellent agreement. Besides, in all figures, solid lines explain the first (UBS) solution, whereas the dotted lines display the second (LBS) solution.

**Table 1:** Thermo physical properties of base fluid and ${\text{Ti}}_{{6}} {\text{Al}}_{{4}} {\text{V}}$ (Makinde et al.^49^).

Material	Water	${\text{Ti}}_{{6}} {\text{Al}}_{{4}} {\text{V}}$
$C_{p} \left( {{\text{J}}/{\text{kgK}}} \right)$	4179	0.56
$\rho \left( {{\text{kg}}/{\text{m}}^{3} } \right)$	997.1	4420
$k\left( {{\text{W}}/{\text{mK}}} \right)$	0.613	7.2
$\sigma \left( {{\text{S}}/{\text{m}}} \right)$	0.005	$5.8 \times 10^{6}$
Pr	6.2	–

**Table 2:** Assessment of the values of $F^{\prime\prime}\left( c \right)$ when $\phi = M = We = 0$.

$c$	$\lambda = 0$		$\lambda = - 1$		$\lambda = - 1$	
	Soid et al.^8^	Present	Soid et al.^8^ Present		Present	
			First solution	Second solution	First solution	Second solution
0.01	8.491454	8.4915	26.599394	2.805533	26.6021	2.8031
0.1	1.288778	1.2888	3.703713	0.389103	3.7162	0.3884
0.2	0.751665	0.7515	2.005424	0.227837	2.0055	0.2278

**Table 3:** Assessment of the values of $F^{\prime\prime}\left( c \right)$ for different values of $\phi$ when $\phi = 0.1,We = 0.05,$ $\lambda = - 3.5,c = 0.1$.

$\phi$	bvp4c		Keller-box	
	First solution	Second solution	First solution	Second solution
0	1.2173	0.6990	1.2196	0.6999
0.03	1.3028	0.7688	1.3052	0.7694
0.06	1.4028	0.8399	1.4066	0.8401
0.1	1.5635	1.9361	1.5651	1.9381

### Deviation of the skin coefficient and the local Nusselt number

Figures 2 and 3 present the deviation of the skin friction ${\text{Re}}_{{x_{1} }}^{1/2} C_{f}$ and the Nusselt ${\text{Re}}_{{x_{1} }}^{ - 1/2} Nu_{{x_{1} }}$ for varying nanoparticle volume fraction $\phi$ versus ratio velocity parameter $\lambda$. It is scrutinized from Fig. 2 that the increment in the value of $\phi$ as a result the UBS, as well as LBS, augment. This is because that the nanoparticles thermal conductivity becomes larger which causes more suspension of particles in the host fluid. Figure 3 reveals that the values of ${\text{Re}}_{{x_{1} }}^{ - 1/2} Nu_{{x_{1} }}$ confirming and to show the escalating behavior in the LBS, as well as the UBS owing to the large value of $\phi$. However, the multiple results are existing in the regions of $\lambda_{c} < \lambda < 0$ while a single result of the phenomenon of $\lambda = \lambda_{c} < 0$ and in the case $\lambda > 0$ (the free-stream as well as the needle travel in the identical direction). It is observed from our calculation that the critical points $\lambda_{c}$ are -3.8448, -3.7700, and -3.7670 for $\phi = 0,0.05,1$, respectively. It is concluded from critical values that the separation of boundary delays in the absence of the nanoparticle $\left( {\phi = 0} \right)$. Figures 4 and 5 discussed the behavior of Weissenberg number $We$ on ${\text{Re}}_{{x_{1} }}^{1/2} C_{f}$ and ${\text{Re}}_{{x_{1} }}^{ - 1/2} Nu_{{x_{1} }}$, respectively. These figures suggest the decreasing tendency in the LBS as well as UBS for augmenting the value of $We$. Here, the critical values in the case of the Weissenberg effect are − 3.8760, − 3.8650 and − 3.8600 for $We = 0,0.03,0.05$, respectively. Impacts of radiation, and heating parameters on ${\text{Re}}_{{x_{1} }}^{1/2} C_{f}$ and ${\text{Re}}_{{x_{1} }}^{ - 1/2} Nu_{{x_{1} }}$ are portrayed in Figs. 6 and 7, respectively. Figure 6 depicts that the outputs of the Nusselt number enhance in the LBS, and UBS with the augmentation in the radiation parameter. Due to the influence of radiation, the critical values are obtained as − 3.8610, − 3.8605, and − 3.8605 for the three selected values of $R_{d} = 0.1,0.3,0.5$, respectively. On the other hand, the impact of the heating parameter in Fig. 7 is quite different (like Fig. 6). More precisely, the values of the Nusselt parameter show a decelerated behavior in the UBS and escalating in the LBS owing to upsurges the value of the heating parameter. In this case, the established critical values are the following − 3.8610, − 3.8600, and − 3.8600 for $\theta_{r} = 0.1,0.3,0.5$, respectively. The trajectories of ${\text{Re}}_{{x_{1} }}^{1/2} C_{f}$ and ${\text{Re}}_{{x_{1} }}^{ - 1/2} Nu_{{x_{1} }}$ for selected values of $M$ are demonstrated in Figs. 8 and 9. The graph in Fig. 8 confirms that the values of ${\text{Re}}_{{x_{1} }}^{1/2} C_{f}$ behaving increasingly in the UBS and contrary in the LBS. However, the contrasting behavior is scrutinized for ${\text{Re}}_{{x_{1} }}^{ - 1/2} Nu_{{x_{1} }}$ as depicted in Fig. 9. The critical values are − 4.2760, − 4.0240, and − 3.8610 for $M = 0,0.03,0.05$, respectively. Thus, the critical values $\left| {\lambda_{c} } \right|$ decrease, suggesting that the magnetic parameter rises the separation of the boundary.

### Deviation of the velocity and temperature fields

The impact of the Weissenberg parameter $We$ on fluid velocity and temperature distribution is shown in Figs. 10 and 11, respectively. Figure 10 displays that the fluid velocity shrinks with augmenting $We$ in the stable and unstable branch solutions which in turn enhances the thickness of the velocity BLF. Since the Williamson parameter is the ratio of relaxation to retardation time. Therefore, uplifting values of $We$ upsurges the relaxation time. Due to this, the liquid particles need extra time to reinstate their original path. However, the temperature distribution decline with escalating values of $We$ in the upper branch solution, whereas the opposite behavior is seen in the lower branch solution as depicted in Fig. 11. The influence of needle size $c$ on the fluid velocity is depicted in Fig. 12. It is visible that growing the size of the needle disturbs the free-stream, which consecutively causes to moderate the velocity of the fluid in the UBS, while the liquid velocity increases in the LBS. Thus, the magnitude of the momentum boundary-layer increases in the upper solution and shrinks in the lower solution. Thus, the boundary thickness can be organized via the needle size. In Figs. 13 and 14 show the behavior of the magnetic field $M$ on the velocity and temperature profiles, respectively. Figure 13 depicts that the liquid velocity shrinks with increasing $M$ in the UBS and consequently enhances the field of velocity gradient and the magnitude of the boundary layer. On the other hand, the contrary behavior is noticed in the LBS. In physical point of view, the accumulation of a magnetic field will convey a kind of resistive force known as the Lorenz or drag force, which has a tremendous property to reduce or stop the liquid velocity. Figure 14 establishes that the temperature profile upsurges in the UBS and diminutions in the LBS for the larger values of $M$. Physically, a Lorentz force is created by the applied magnetic field which has an important role to delay the motion of liquid, and consequently, the thermal boundary layer and temperature enhances. Thus, needle temperature can be managed by managing the magnetic field strength. Figure 15 shows the patterns of streamlines for nanofluid. The patterns illustrate that the streamlines are more obscured and divided into two regions owing to due to reverse the direction of free stream and thin needle.

## Main remarks

This paper scrutinized the impact of nonlinear radiation on MHD flow with the characteristic of heat transfer suspended in Williamson fluid comprising titanium alloy nanoparticle through a thin needle. The system of arising PDEs is altered into the highly nonlinear ODEs through similarity variables and then functioned out numerically via the built-in function in Matlab called bvp4c. The dual nature of solutions is found for the current problem where the results are shown in both tabular and graphical form (sees physical explanation). The influences of sundry constraints on the flow field are elaborated with the assistance of plots. Also, the stability analysis is highlighted in the presented work to check which solutions are physically stable or not. The vital outcomes of the paper are summarized as the following:

- The rate of heat transfer as well as the skin friction augments due to nanoparticle volume fraction in both UB and LB solutions.
- The liquid velocity gradient is a decaying function of the Weissenberg number in both solutions, while the liquid temperature declines in UBS and upsurges in LBS.
- The rate of heat transfer increases due to the radiation parameter in both branches of solutions.
- Multiple solutions are achieved to survive when free stream and needle shows their progress in conflicting directions whilst the result is unique when they move in the same direction.
- The heat transfer rate declines in the UBS and augments in the LBS as we upsurge the impact of heating parameter.
- The skin friction is decelerating in the UBS and as well as in the LBS owing to the implementation of the larger values of the Weissenberg number while the same behavior is perceived for the rate of heat transfer.
- The skin friction augments due to the magnetic field in UBS and diminishes in the LBS, where the reverse impact is seen in the heat transfer rate.

## List of symbols

${\text{A}}_{{1}}$: First tensor of Rivlin–Erickson
$B$: Magnetic field (kgs^−^^2^ A^−^^1^)
$C_{f}$: Skin friction coefficient
$${\text{Nu}}_{{x_{1} }}$$: Nusselt number
$B_{0}$: Strength of the magnetic field (A/m)
$c$: Size of the needle
$$M$$: Magnetic parameter
$$\Pr$$: Prandtl number
$${\rm I}$$: Identity vector
$k^{*}$: Coefficient of mean absorption
*k*: Thermal conductivity (Wm^−^^1^ K^−^^1^)
$q_{w}$: Heat-flux (Wm^−^^2^)
$F$: Similarity fuction for velocity
${\text{Re}}_{{x_{1} }} ,{\text{Re}}_{{r_{1} }}$: Local Reynolds number
$$R_{d}$$: Radiation parameter
${\text{S}}_{1}$: Extra stress-tensor
$$T_{1}$$: Temperature (K)
$T_{w}$: Temperature of the wall (K)
$$T_{\infty }$$: Ambient temperature (K)
$$u_{w}$$: Velocity (m/s)
$u_{\infty }$: Free-stream velocity (m/s)
$$\left( {u_{1} ,v_{1} } \right)$$: Velocity components (m/s)
$$We$$: Weissenberg number
$\left( {r_{1} ,x_{1} } \right)$: Radial and axial directions (m)

## Greek symbols

$\lambda$: Velocity ratio parameter
$\nu_{f}$: Kinematic viscosity of the base fluid (m^2^/s)
$$\alpha_{f}$$: Thermal diffusivity (m^2^/s)
$\mu_{0} ,\mu_{\infty }$: Limiting viscosities at zero and infinite shear stresses (Nsm^−^^2^)
$$\dot{\gamma }_{1}$$: Shear stress
$$\theta$$: Dimensionless temperature
$\theta_{r}$: Temperature ratio parameter
$\phi$: Volume fraction of nanoliquid
$\sigma$: Electric conductivity of nanofluids (Sm^−^^1^)
$\sigma^{*}$: Stefan Boltzmann constant (Wm^−^^2^ K^−^^4^)
$\rho$: Nanofluid density (kg m^−^^3^)
$\mu$: Viscosity of nanoliquid (Kg m^−^^1^ s^−^^1^)
$\left( {\rho c_{p} } \right)$: Heat capacity of nanoliquid (J/K)
$$\Gamma_{1}$$: Time relaxation (s)
$$\psi_{1}$$: Stream function
$$\eta$$: Similarity variable
$\tau_{w}$: Wall shear stress

## Subscript

nanof: Nanofluid
f: Base fluid
